# Comparison of spheno-occipital synchondrosis maturation stages with three-dimensional assessment of mandibular growth

**DOI:** 10.1186/s12903-022-02692-3

**Published:** 2022-12-30

**Authors:** Waseem S. Al-Gumaei, Reem Al-Attab, Barakat Al-Tayar, Saba A. Al-hadad, Enas S. Alyafrusee, Abeer A. Al-mashraqi, Najah Alhashimi, Yan Zheng, Maged S. Alhammadi

**Affiliations:** 1 Department of Orthodontics and Dentofacial Orthopedics, School of Stomatology, Lanzhou University, Lanzhou, China; 2 Department of the Dental Implant, School of Stomatology, Lanzhou University, Lanzhou, China; 3 Department of Pre-Clinical Oral Health Sciences, College of Dental Medicine, QU Health, Qatar University, Doha, Qatar; 4 Unit and Divisional Chief Orthodontics at Hamad Medical Corporation, College of Dental Medicine, Qatar University, Doha, Qatar; 5 Orthodontics and Dentofacial Orthopedics, Department of Preventive Dental Sciences, College of Dentistry, Jazan University, Jazan, Saudi Arabia

**Keywords:** Mandibular growth, Spheno-occipital synchondrosis, Stages of fusion, CBCT, Three-dimensional

## Abstract

**Background:**

This study aimed to compare spheno-occipital synchondrosis (SOS) maturation stages with a three-dimensional assessment of mandibular growth.

**Methods:**

This is a cross-sectional study of a retrospective type, in which cone-beam computed tomography (CBCT) images of 500 patients aged 6 to 25 years (226 males and 274 females) were analyzed. The SOS was evaluated using the four-stage scoring system; completely open, partially fused, semi-fused, or completely fused. The SOS scoring and three-dimensional cephalometric measurements were analyzed by Invivo 6.0.3 software. Descriptive and analytical statistics were performed, and a *P*-value < 0.05 was considered statistically significant.

**Results:**

There was a statistically significant difference in mandibular measurements among SOS maturation stages in both sexes (*P* < 0.05). The skeletal growth increments of mandibular variables across the SOS stages had higher mean differences between SOS stages 2 and 3 than those between stages 1 and 2 and stages 3 and 4 in both sexes. The mandibular growth curves increased with chronological age (earlier in females) and SOS maturation stages (mostly in stages 1, 2, and 3 than stage 4).

**Conclusions:**

The SOS maturation stages are valid and reliable mandibular skeletal indicators as evaluated with three-dimensional cephalometric mandibular measurements. The findings of growth increments and constructed growth curves of mandibular growth might be helpful in diagnosis and treatment planning.

**Supplementary Information:**

The online version contains supplementary material available at 10.1186/s12903-022-02692-3.

## Introduction

The spheno-occipital synchondrosis (SOS) is located in the midline between the sphenoid and occipital bones. It is considered the most important growth center in the cranial base because of its late ossification and contribution to post-natal cranial base growth [1, 2]. The cranial base is the template for facial development; therefore, it is directly related to the maxillary and mandibular growth and displacement. In individuals with craniofacial syndromes like Apert, Crouzon, Down, or Pfeiffer syndromes, the SOSs showed early ossification correlated with a shorter cranial base and midface hypoplasia [3, 4].

The evaluation of craniofacial skeletal growth has critical importance in orthodontic, dentofacial orthopedic, orthognathic diagnosis, treatment planning, and evaluation of treatment result's prognosis and stability [5, 6]. The main area of interest for the orthodontist is to know whether a patient has attained peak pubertal growth or passed that point. This, in turn, determines whether growth modification is still a viable treatment option [7, 8].

The most commonly used craniofacial skeletal maturation indicators were hand-wrist (HW) and cervical vertebrae maturation (CVM) methods. However, each method has its inherited limitations [9, 10]. The hand-wrist method requires expert knowledge and expenditure of time by the operator, the method's accuracy is not very high, and it exposes the patient to an unnecessary radiation dose [11]. The CVM method possesses poor reproducibility attributed to the level of training, clinician experience, and assessment methods [12–14]. Furthermore, the CVM method could not predict the amount of craniofacial growth in girls with Class II malocclusion [15, 16]. It is generally believed in the orthodontic community that there is still a need for a reliable skeletal maturity indicator that shows efficacy in detecting mandibular growth and should not depend on only one skeletal indicator for clinical decisions [17–19]. Based on recent high level evidence the CVMI and the HW radiograph still not guarantee to provide a reliable tool for skeletal age assessment and it was recommended that further studies are warranted to confirm these findings or to validate another more effective tool and it was also suggested to use a combination of maturation signs along with development stages of cervical vertebrae in order to determine skeletal maturation until a quantitative and valid method is presented [11, 20].

The CBCT images provide accurate three-dimensional anatomic details and facilitate visualization of small osseous structures and high-resolution images compared to conventional radiographs [21]. Recently, the SOS method has been considered a valid and reliable indicator of skeletal age compared with the CVM, HW methods, and chronological age [10, 22–26]. Jabour studied mandibular growth and the SOS fusion stages, but he used two-dimensional lateral cephalometric radiographs (LC) to measure the total mandibular length [22]. Up to date, the available literature neither compared the SOS maturation stages with the three-dimensional growth of the mandible nor included the reliability between them. Therefore, this study aimed to evaluate three-dimensional mandibular growth during SOS fusion stages in both sexes, assessing the reliability of the SOS method as a skeletal indicator of 3D mandibular growth, calculating mandibular growth potential (growth increments), and constructing a mandibular growth curve.

## Materials and methods

### Sample selection

This cross-sectional study of a retrospective type was approved by the Ethics Committee of the School of Stomatology at Lanzhou University in a group of the Chinese population (No: LZUKQ-2019-042). The sample size primarily depended on previous studies [27, 28], the G* power 3.0.10 software (ver. 3.1.9.7; Heinrich-Heine-Universität Düsseldorf, Düsseldorf, Germany) was used to calculate the sample size based on the length of mandible (the primary outcome of this study). The a priori sample size estimation, performed at a 5% level of significance (α = 0.05), with a power of 99%, with mean vales of length of mandible were 96.27 ± 4.95 in SOS stage I and 101.57 ± 5.39 in SOS stage II, effect sizes (d = 1.02), and a two-sided test comparing two independent samples. The calculation revealed that a minimum of 37 subjects were necessary per SOS stage group (four group for each sex).

Data were randomly collected based on the pre-existing records between January 2016 and July 2021 according to a known patient age, sex, dental and medical history, and CBCT scan*.* The inclusion criteria were (1) age range from 6 to 25 years in which the upper and lower limits were determined following previous studies [29–31]; and (2) clear reporting of sex, dental and medical history. Exclusion criteria included (1) patients with reported cleft lip or cleft palate [32]; (2) craniofacial syndromes; (3) head trauma and/or deformity; (4) gross asymmetry; (5) previous orthodontic or orthopedic treatment, or (6) inadequate diagnostic quality radiographs. The huge amount of data in the institutional data-base were retrieved and analyzed for the preset selection criteria. Most of the retrieved CBCT were taken as per the institutional policy upon patients’ registration that comprehensive investigations to exclude and/or assess any pathology in the craniofacial region (delayed eruption teeth, root resorption, whole survey dentition, third molar extraction, bone anomaly, impacted teeth, TMJ, construction customized maxillary speed expansion (MSE), and diagnosis of nasal-complex problems) and clearly mentioned the benefits and possible risks of CBCT giving the condition that the patient has not been referred for CBCT in the last 6 months. The information of 572 subjects were collected, of which 72 were excluded, the remaining five hundred subjects, 274 females and 226 males were included in this study. Sample grouping was based on SOS scoring into four groups for each sex.

Informed consent was obtained from all subjects and their parents or legal guardians. Moreover, all methods were carried out in accordance with the principles of the declaration of Helsinki.

### Three-dimensional imaging

#### CBCT acquisition

I-CAT Imaging System (Imaging Sciences International Inc. Hatfield, U.S.A.) was used to acquire CBCT images. Each patient had a scan utilizing a standard technique that comprised a standard head position, maximum intercuspation with the Frankfort horizontal plane parallel to the floor, and a crossing laser guide. Based on the imaging protocol, the patient was warned neither to swallow nor move throughout the scanning process[33]. The parameters of acquisition were16 × 13 cm field of view, 120 kV, 18.54 mAs, and 8.9 s exposure time. The selected slice thickness was 2 mm, and the voxel dimension was 0.3 mm[26].

#### SOS fusion staging

**Fig. 1:**
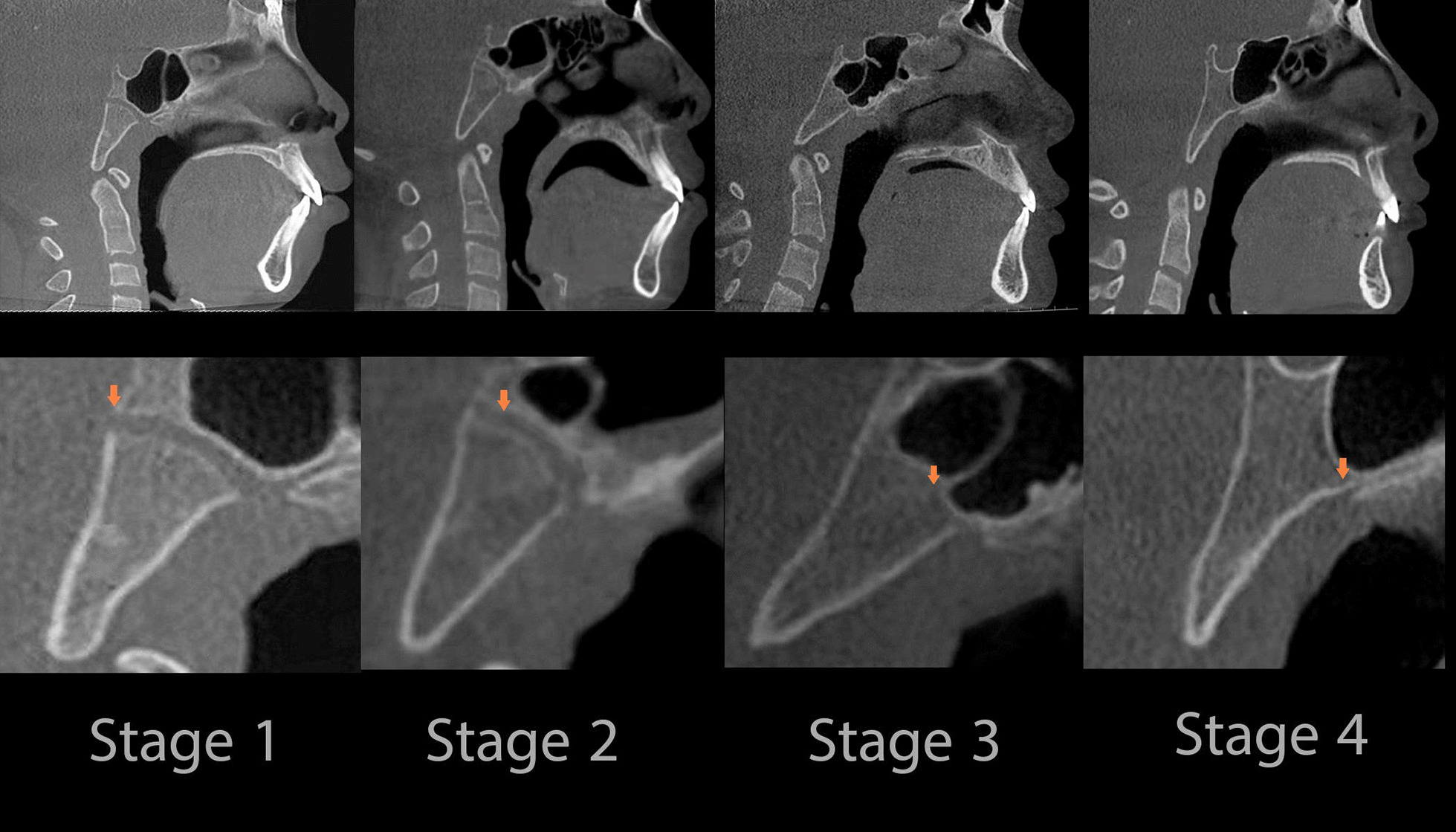
Stages of SOS fusion in the mid-sagittal plane (3D multi-planar reconstruction)

**Fig. 2:**
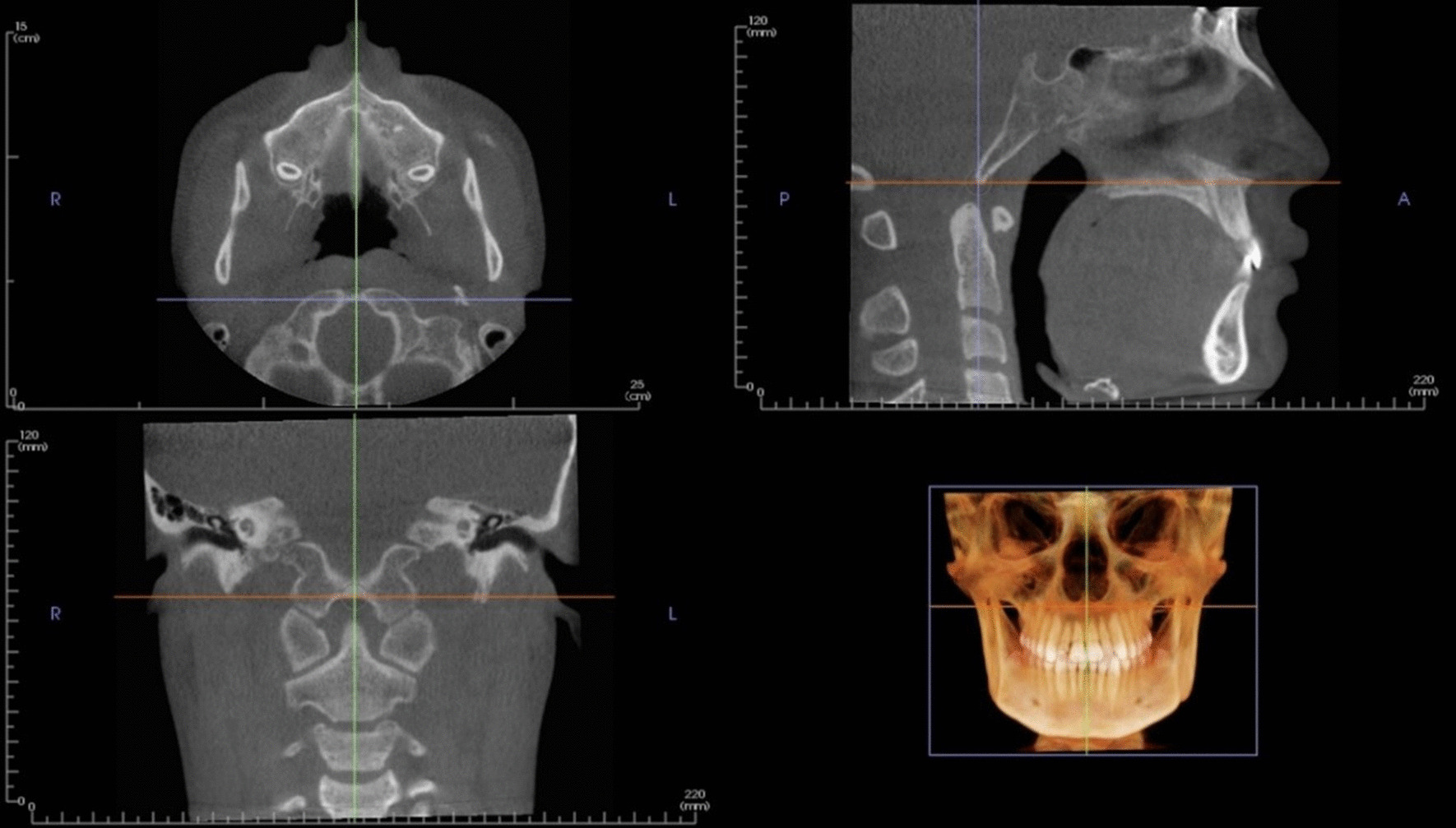
Mid-sagittal CBCT evaluation of spheno-occipital synchondrosis with the head in the default orientation

Digital Imaging and Communications in Medicine (DICOM) files of the CBCT images were obtained and then imported into Invivo 6.0.3 software (Anatomage, San Jose, CA, USA). The spheno-occipital synchondrosis four-stages system of Franklin and Flavell [29] (Table 1, Fig. 1) was followed. Lottering et al.[34] 6-stage SOS scoring system assumes the presence of fusion scar, which might persist for decades after fusion, has occurred [35]. Moreover, the four-stage scoring approach reduces assessment subjectivism, resulting in increased inter-observer agreement [29]. Moreover, it may be easier and needs less training by clinician and recommended by previous studies [24, 29, 30, 36, 37]. All 3D virtual models were oriented at a standardized position, then adjusted to the mid-sagittal plane (MSP) view (Fig. 2) [24, 29–31, 38, 39]. All CBCTs were assessed blindly with a coding system to mask the patient's demographic data and recorded in a separate data extraction sheet. Two well-trained observers, WA and RA independently scored the entire sample. Separated by a 1-month interval, both observers randomly selected 100 images and re-evaluated for intra-observer and inter-observer agreement of SOS staging. In the cases of disagreement, the axial view was used to assess the synchondrosis to reach a consensus as recommended by Okamoto et al.[40].

**Table 1:** Description of the fusion of the spheno-occipital synchondrosis scoring

Stage	Status	Description
1	Un-fused	Opened entirely with no sign of closure or presence of bone in the gap between the endocranial and ectocranial borders
2	Partial-fused	Fused endocranially but not more than half the length of the synchondrosis (Fusing endocranially, ≤ 50%)
3	Semi-fused	Fusing ectocranially with more than half the length of the synchondrosis but without fusing of the inferior (ectocranial) border. (Fusing ectocranially, > 50% and less than 100% fusion)
4	Complete fusion	Fused entirely with normal bone appearance throughout the synchondrosis, but a fusion scar may exist

#### Three-dimensional measurements

**Fig. 3:**
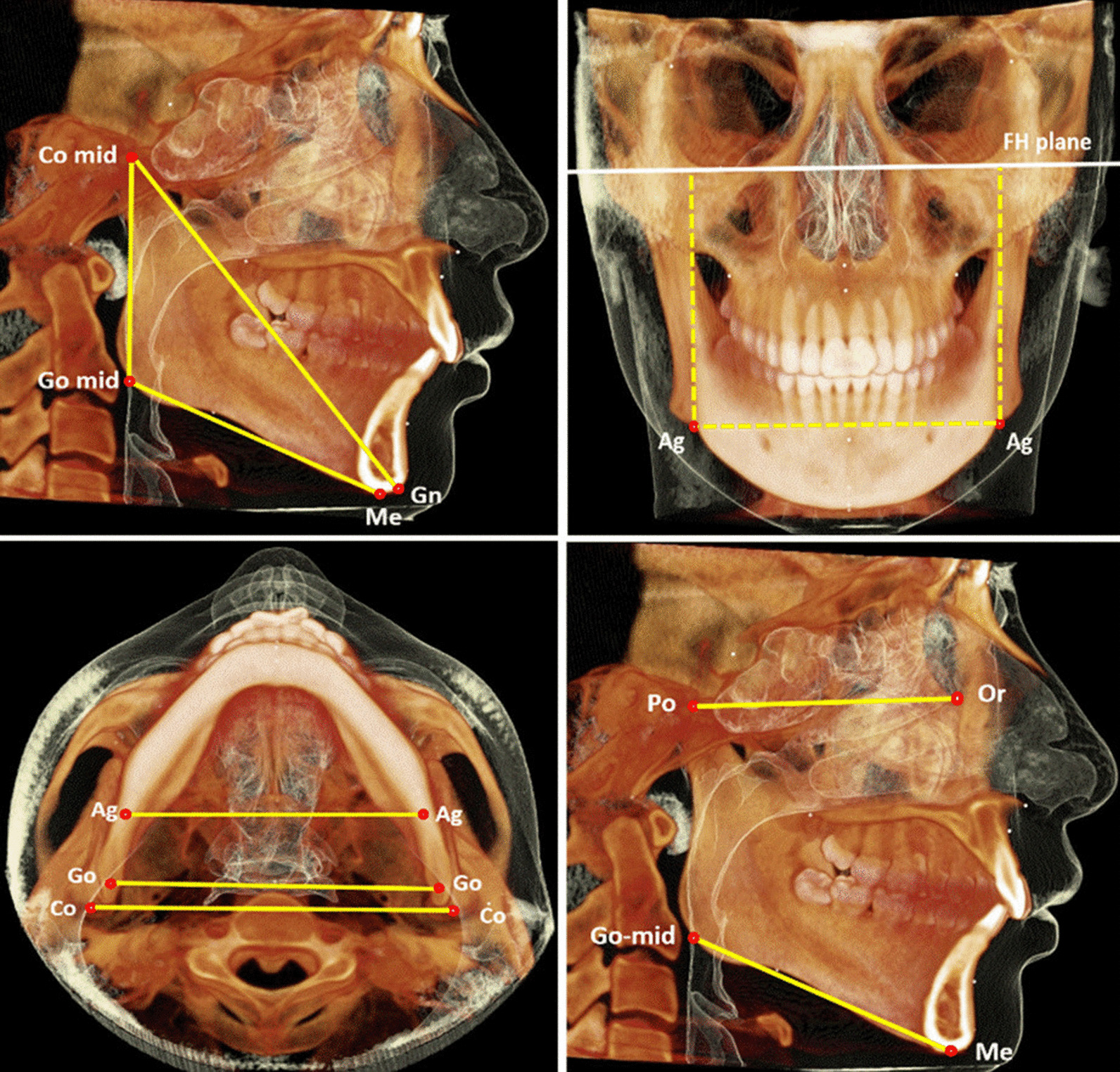
Three-dimensional cephalometric measurements of the mandible

The 3D analysis involved the identification of anatomical landmarks (see Additional file 1: Table S1), reference planes (see Additional file 1: Table S2), and 3D linear and angular measurements presented in Table 2 and graphically presented in Fig. 3 [27, 41, 42].

**Table 2:** Definitions of the three-dimensional skeletal measurements of mandible

Measurement	Abbreviation	Definition
Mandibular total length	Co mid-Gn (mm)	Distance between Co midpoint and Gn points onto the sagittal plane
Mandibular body length	Go mid-Me (mm)	Distance between Go midpoint and points Me point onto the sagittal plane
Mandibular ramal height	Co mid-Go mid (mm)	Distance between Co midpoint and Go midpoint points onto the sagittal plane
Mandibular width Biantegonial width	Ag–Ag (mm)	Distance between the right and the left Ag points along the transverse axis
Mandibular width Bigonial width	Go–Go (mm)	Distance between the right and the left Go points along the transverse axis
Mandibular width Bicondylar width	Co–Co (mm)	Distance between the right and the left Co points along the transverse axis
Mandibular height	Ag–FHP (mm)	Distance between the left and the right Ag points and FH plane along the transverse axis
Mandibular anterio-posteiorly inclination	(MP mid/FHP)°	The angle between of Go mid-point-Me line and the FHP anterio-posteriorly onto the sagittal plane
Mandibular medio-lateraly inclination	(Ag–Ag/FHP)°	The angle between of mandibular plane Ag–Ag line and FHP medio-lateraly onto the coronal plane

With the nasion point centered as the origin of the 3D mold at the center of three planes X, Y, and Z (coronal, axial, sagittal), then was calculated by the 3D equation of distance formula to provide a more reliable and accurate measuring:

$$d = \sqrt {\left( {{\text{x1 }}{-}{\text{ x2}}} \right){2 } + { }\left( {{\text{y1 }}{-}{\text{ y2}}} \right){2 } + { }\left( {{\text{z1 }}{-}{\text{ z2}}} \right){2}}$$

where d is the distance (in millimeters) between two anatomic landmarks, and × 1, y1, and z1 and × 2, y2, and z2 are the coordinates of the two landmarks at the two ends of the linear measurement.

Intra- and inter-observer reliability of three-dimensional measurements was assessed by re-measuring 10% of the sample (50 CBCTs) by two observers (W.A and R.A) at one-month intervals.

### Statistical analysis

IBM SPSS Statistics for Windows, Version 26.0 (Armonk, NY: IBM Corp.), was used. Intra- and inter-observer reliability analysis for the SOS scoring was calculated using Cohen's Kappa coefficient [26]. In contrast, the three-dimensional measurements' reliability was calculated by absolute and relative technical measurement errors (TEM and RTEM) and Interclass Correlation Coefficient (ICC) test. Descriptive statistics, including each variable's mean and standard deviation, were calculated and presented. Dahlberg's formula was also used to calculate the Standard Deviation of Measurement Error (SE) [43]. Quantitative data for the normal state was explored by the verification distribution of data. Depending on Shapiro–Wilk test and Kolmogorov–Smirnov test, all groups showed a normal distribution. The data were presented as mean and standard deviation (SD) for comparative analysis.

One-way ANOVA test was used to compare between the SOS maturation stages (four SOS groups per sex: independent variables) regarding the linear and angular measures of the mandible (dependent variables) for males and females separately. Two-way ANOVA was used to compare SOS’s four groups and sex (independent variables) regarding the linear and angular measures of the mandible (dependent variables). The problem of comparisons was treated by using Bonferroni correction, adjusting the P-value for multiple comparison tests, to avoid type I error.

The growth curves for mandibular parameters based on SOS maturation stages and chronological age were determined following a previous study [44]. R-statistical programing language was used for graphing and computing R^2^. *P*-value < 0.05 was considered statistically significant.

## Results

CBCT scans of 500 patients aged 6 to 25 years; with a mean age of 13.89 ± 1.13 years were analyzed. It included 274 females and 226 males with mean ages of 13.68 ± 5.30 and 14.14 ± 4.99 years, respectively. The results of the intra- and inter-examiner reliability analysis for the SOS scoring were "almost perfect"; weighted Kappa agreement measures were more than 0.900 for each observer (see Additional file 1: Table S3). Three-dimensional mandibular cephalometric measurement's reliability was "excellent agreement"; R* values of TEM and RTEM were higher than 0.95% and ICC above 98% with *P* < 0.05 (see Additional file 1: Table S4).

The results showed that there were statistically significant differences in millimetric mandibular measurements (Co–Gn, Go–Me, Ag–Ag, Go–Go, Co–Co, Co–Go, Ag-R/FHP, and Ag-L/FHP), as well as the angular measurements (MP/FHP and Ag–Ag/FHP) for both sexes as presented in Table 3. The pair-wise comparison showed differences across different SOS stages. In contrast, the two-way ANOVA results showed no statistically significant difference in all used mandibular measurements according to sex and SOS stages interactions except for these parameters; Go–Go, Co–Co, Co–Go, Ag-R/FHP, and Ag-L/FHP.

**Table 3:** Descriptive statistics and the results of the One-way ANOVA test (for males and females separately) and two-way ANOVA test (for both sexes) between SOS fusion stages and 3D measurements of mandibular growth pattern

Measurements	Sex	Stage 1	Stage 2	Stage 3	Stage 4	SOS groups Comparison One-way ANOVA	SOS*Sex Comparison two-way ANOVA
		Mean ± SD	Mean ± SD	Mean ± SD	Mean ± SD	*p* value	*p* value
Co mid-Gn (mm)	M	96.27 ± 4.95^a^	101.57 ± 5.39^b^	109.34 ± 6.47^c^	114.51 ± 6.16^d^	0.000**	0.120
	F	91.44 ± 5.53^a^	96.10 ± 5.05^b^	102.91 ± 4.41^c^	106.76 ± 4.29^d^	0.000**	
Go mid-Me (mm)	M	59.21 ± 3.17^a^	62.38 ± 3.62^b^	67.63 ± 4.46^c^	71.54 ± 4.61^d^	0.000**	0.101
	F	57.02 ± 4.13^a^	59.62 ± 3.82^b^	63.75 ± 3.66^c^	67.11 ± 3.72^d^	0.000**	
Ag-R-Ag-L (mm)	M	77.03 ± 4.23^a^	80.69 ± 4.21^b^	86.17 ± 4.22^ cd^	87.10 ± 4.75^d^	0.000**	0.057
	F	72.83 ± 4.48^a^	75.57 ± 3.83^b^	79.45 ± 3.81^c^	82.93 ± 3.63^d^	0.000**	
Go–Go (mm)	M	83.84 ± 5.61^a^	88.38 ± 4.79^b^	95.24 ± 6.33^cd^	96.09 ± 5.66^d^	0.000**	0.033*
	F	79.50 ± 4.73^a^	82.35 ± 4.81^b^	87.19 ± 4.88^c^	91.31 ± 4.86^d^	0.000**	
Co–Co (mm)	M	92.36 ± 4.37^a^	96.36 ± 4.45^b^	102.87 ± 5.33^c^	105.40 ± 5.45^d^	0.000**	0.005*
	F	89.89 ± 4.22^a^	91.90 ± 3.89^a^	95.82 ± 4.33^b^	100.22 ± 4.81^c^	0.000**	
Co mid-Go mid (mm)	M	47.52 ± 4.21^a^	51.36 ± 4.39^b^	57.31 ± 5.43^c^	61.30 ± 4.70^d^	0.000**	0.025*
	F	44.52 ± 2.89^a^	47.85 ± 3.38^b^	51.64 ± 3.14^c^	55.89 ± 3.82^d^	0.000**	
Ag-R/FHP (mm)	M	56.27 ± 4.38^a^	61.44 ± 4.26^b^	68.20 ± 5.41^c^	72.74 ± 4.41^d^	0.000**	0.013*
	F	53.26 ± 4.03^a^	57.08 ± 3.65^b^	61.92 ± 3.67^c^	66.78 ± 3.95^d^	0.000**	
Ag-L/FHP (mm)	M	55.77 ± 4.73^a^	60.48 ± 4.47^b^	67.52 ± 5.64^c^	71.13 ± 6.43^d^	0.000**	0.048*
	F	52.66 ± 3.85^a^	56.19 ± 3.60^b^	61.03 ± 3.73^c^	66.21 ± 4.26^d^	0.000**	
(MP mid-FHP)°	M	28.22 ± 5.18^a^	27.41 ± 5.06^ab^	24.91 ± 5.89^b^	21.83 ± 5.56^c^	0.000**	0.217
	F	28.90 ± 4.89^a^	28.13 ± 4.49^a^	27.77 ± 4.50^a^	24.35 ± 5.16^b^	0.000**	
(Ag–Ag/FHP)°	M	0.78 ± 0.68^a^	1.01 ± 0.76^a^	0.94 ± 0.75^ab^	1.10 ± 0.80^b^	0.003**	0.481
	F	0.76 ± 0.68^a^	0.89 ± 0.69^a^	1.00 ± 0.69^ab^	1.27 ± 1.03^b^	0.002**	

*Indicate significance at the 0.05 level (2−tailed)

**Indicate significance at the 0.01 level (2−tailed)

^a,b,c,d^Superscripts in the same row represent a statistically significant difference between SOS stages according to multiple comparisons of Bonferroni Post hoc analysis

**Fig. 4:**
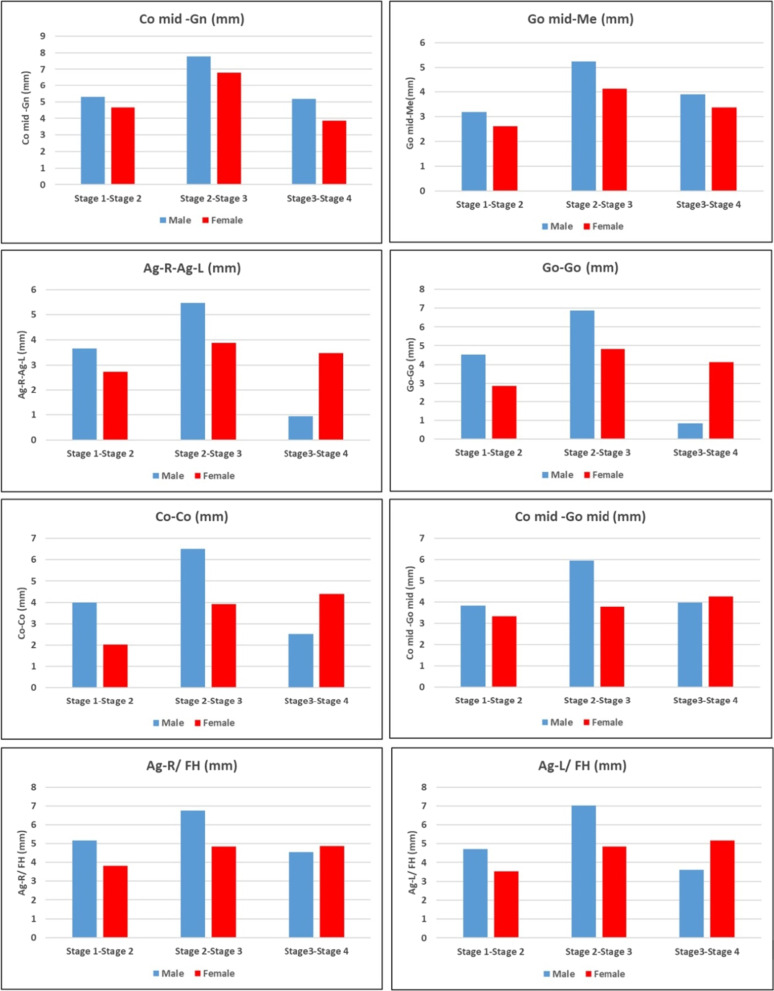
The growth increments of the mandible across the SOS maturation stages

Regarding the skeletal growth increments (mean differences) of the mandible among the SOS stages (Table 4, Fig. 4); the results showed there were statistically significant mean differences between SOS stages 2 and 3 that were larger than those between stages 1 and 2 and stages 3 and 4 in males and females for mandibular measurements in millimetric (Co–Gn, Go–Me, Ag-R-Ag-L, Go–Go, Co–Co in males, Co–Go, Ag-R/FHP & Ag-L/FHP in males). However, mean differences between SOS stages 3 and 4 were larger than those between stages 1 and 2 and stages 2 and 3 (Co–Co, Ag-R/FHP & Ag-L/FHP in females).

**Table 4:** The growth increments of the mandible across the SOS maturation stages

Measurement	Sex	Stage 1-Stage 2			Stage 2-Stage 3			Stage3-Stage 4			*P* value
		Mean diff	CI 95%; Min	CI 95%; Max	Mean diff	CI 95%; Min	CI 95%; Max	Mean diff	CI 95%; Min	CI 95%; Max	
Co mid-Gn (mm)	M	5.30^AB^	2.34	8.27	7.77^BC^	4.80	10.74	5.17^CD^	2.28	8.06	*P* < 0.05*
	F	4.66^AB^	2.18	7.14	6.80^BC^	4.51	9.10	3.85^CD^	1.89	5.82	
Go mid-Me(mm)	M	3.17^AB^	1.09	5.24	5.25^BC^	3.18	7.32	3.90^CD^	1.89	5.92	
	F	2.60^AB^	0.60	4.61	4.13^BC^	2.27	5.98	3.37^CD^	1.79	4.95	
Ag-R-Ag-L (mm)	M	3.66^AB^	1.42	5.91	5.48^BC^	3.23	7.73	0.93^DD^	-1.26	3.11	
	F	2.74^AB^	0.69	4.78	3.88^BC^	1.99	5.77	3.48^CD^	1.87	5.10	
Go–Go (mm)	M	4.54^AB^	1.67	7.40	6.86^BC^	3.99	9.72	0.85^DD^	-1.94	3.64	
	F	2.86^AB^	0.31	5.40	4.83^BC^	2.48	7.19	4.12^CD^	2.10	6.13	
Co–Co (mm)	M	3.99^AB^	1.45	6.53	6.51^BC^	3.97	9.05	2.53^CD^	0.06	5.00	
	F	2.01^AA^	-0.32	4.35	3.92^AB^	1.77	6.08	4.40^BC^	2.56	6.24	
Co mid -Go mid (mm)	M	3.84^AB^	1.43	6.24	5.95^BC^	3.55	8.36	3.98^CD^	1.64	6.32	
	F	3.33^AB^	1.52	5.14	3.79^BC^	2.12	5.46	4.25^CD^	2.82	5.68	
Ag-R/FHP (mm)	M	5.17^AB^	2.81	7.53	6.76^BC^	4.40	9.12	4.54^CD^	2.25	6.84	
	F	3.82^AB^	1.80	5.85	4.84^BC^	2.96	6.71	4.86^CD^	3.26	6.46	
Ag-L/FHP (mm)	M	4.71^AB^	1.93	7.50	7.03^BC^	4.25	9.82	3.61^CD^	0.90	6.32	
	F	3.53^AB^	1.45	5.60	4.85^BC^	2.92	6.77	5.17^CD^	3.53	6.82	

*Indicate significance at the 0.05 level (2-tailed)

^A,B,C,D^Superscripts in the same row represent a statistically significant difference between SOS stages according to multiple comparisons of Bonferroni Post hoc analysis

**Fig. 5:**
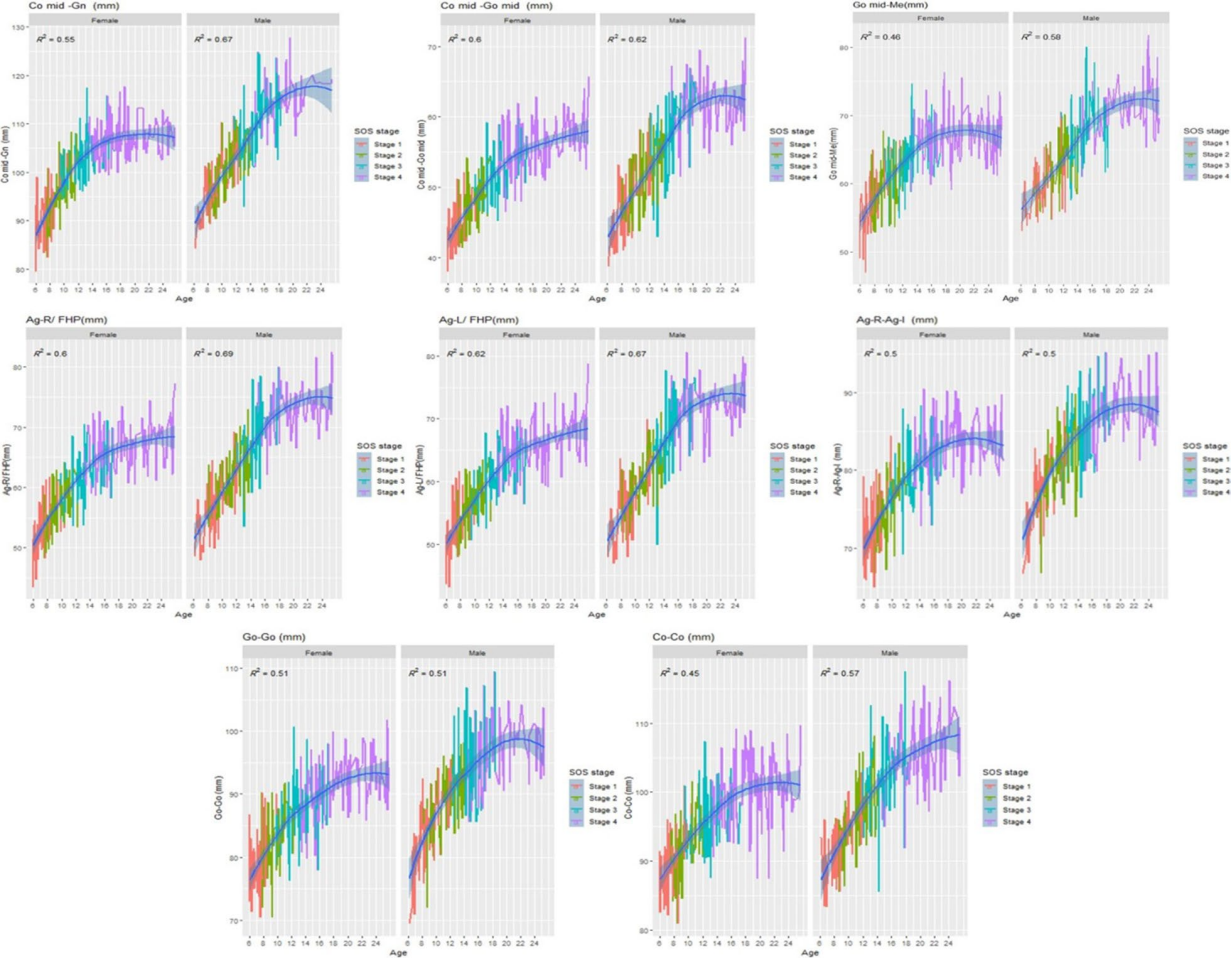
Female and male mandibular growth curves according to the SOS maturation (stages 1–4) and chronological age (6 through 25 years)

The mandibular growth curves according to SOS stages and chronological age for females and males with the effect size R^2^ and *P* < 0.05 are graphically presented in Fig. 5.

## Discussion

The evaluation of skeletal maturation of craniofacial complex has critical importance in orthodontic, dentofacial orthopedic, orthognathic diagnosis, treatment, and prognosis. Recently, the SOS method has been considered as a reliable tool and correlated well with other established methods; Hand-wrist maturation and CVM index in assessment of skeletal age [10, 18, 22, 23, 25, 45–49]. However, through the literature, only two studies considered the SOS maturation stages and craniofacial morphology, but they neither compared the SOS maturation stages with the three-dimensional growth of the mandible nor included the reliability between them.[22, 27].

The superiority of SOS method may relate to the critical location of SOS in cranial base. In which its late ossification and contribution to post-natal cranial base growth play critical role in facial development[1, 2, 50–52]. Moreover, the CBCT have been used widely in dental field, so the SOS may be considered as suitable method as CBCT provides the benefits of low-cost, high-resolution, accurate three-dimensional imaging (3D) without the risk of increased radiation exposure to the patient, and easy visualization of superimposed bony structures[53]. On other hand, Hand -wrist method requires expert knowledge and expenditure of time by the operator, and their accuracy is not very high. It also had the drawback of unnecessary radiographic dose [11].

There were significant differences in the means of mandibular parameters among SOS stages for males and females as the following: the total mandibular length, body length, width (biantegonial, bigonial, and bicondylar), ramal height, mandibular height, anteroposteior inclination, and medio-lateral inclination. This might indicate that the SOS maturation stages have similar proportional growth increases with the significant mandibular parameters. So, this supports using the SOS stages as a valid method to assess mandibular growth. This finding is comparable with the standard gold method of validity of the CVM method for the assessment of facial growth [18, 48].

There was no significant difference in all used mandibular measurements according to sex and SOS stages interaction except for the bigonial width, bicondylar width, and mandibular height. This might indicate no sexual dimorphism in all analyzed mandibular cephalometric parameters according to SOS maturation stages except for the mentioned variables*.* There was no study that mentioned sexual dimorphism in mandibular parameters growth based on SOS maturation stages.

The calculated growth increments between SOS stage 2 and 3 were larger than those between stages 1 and 2 or stages 3 and 4 in males and females for total mandibular length, mandibular body length, mandibular width (biantegonial, bigonial, the male's bicondylar), ramal length, and the male's mandibular height. This might indicate that the mandibular growth peak is between stages 2 and 3. This is in agreement with Jabour [22], who found that the mandibular length growth peak occurred during the fusing stages (stages 2 and 3). Moreover, this finding is supported by the theory that spheno-occipital synchondrosis begins to fuse around puberty [24, 35]. However, mean differences between SOS stages 3 and 4 were larger than those between stages 1 and 2 and stages 2 and 3 for the female's mandibular height and the female's bicondylar width.

Sato et al. [44] constructed growth curves to predict the total mandibular length using the hand-wrist method. In this study, mandibular growth curves were constructed based on SOS maturation stages which were adjusted by incorporating the SOS maturation stages with smoothed fitted values of mandibular parameters considering the effect size (R^2^) on these parameters. This type of adjusting had not been mentioned in any previous studies [44, 54]. The aim behind this adjustment was to evaluate the effect of SOS maturation stages on the mandibular parameters directly; these curves showed that the following mandibular parameters (the total mandibular length, body length, width, ramal height, and mandibular height) increased with increasing of chronological age and it was earlier in females than males. The increase for these parameters with SOS was in stage 1, 2, and 3 (mostly accelerated in stages 2 and 3) then tended to be steadier in stage 4 for males and females in a similar pattern. These findings indicated that these mandibular parameters are more growing as the SOS is un-fused than in the fused stage (stage 4) for both sexes. This is similar to Jabour’s [22] finding of the mandibular length and SOS fusion stages. Moreover, it supported with idea of using the SOS fusion stages as a biological indicator for craniofacial and mandibular growth spurt prediction[24, 36].

A sexual dimorphism was found in this study as the fusion of SOS and growth of mandible were earlier in females than males. This sexual dimorphism is consistent with previous research, which reported that the SOS fuses earlier in females than males [24, 29, 30]. The effect size (R^2^) of SOS stages on mandibular parameters; the total mandibular length, body length, width (biantegonial, bigonial, and bicondylar), ramal height, and mandibular height were between 45 and 62% for females and between 50 and 62% for males. These percentages represent the variation of these mandibular parameters related to the SOS maturation stages. Previous studies reported that the R^2^ was high for biological data (used the CVM method), ranging from 30 to 67% [13, 55]. This may reflect the applicability of the present study growth curves as their effect sizes of SOS on mandibular parameters were mostly high.

Regarding ethical concerns that CBCT is taken in all cases, the policy of the institution that all patients upon registration should sign an informed consent which included their approval for referral to comprehensive investigations to exclude any pathology in the craniofacial region and clearly mentioned the benefits and possible risks of CBCT giving the condition that the patient has not been referred for CBCT in the last 6 months. In addition, the patients’ need any interventions either orthodontic or surgical; all CBCT images used as pre-treatment records are required for the planned treatment without the need for any other radiographic records. Moreover, this is a retrospective study, and the research team didn’t expose patients to extra radiation for the research purpose as all data were collected retrospectively and the study was also approved by the Ethics Committee of the School of Stomatology at Lanzhou University in a group of the Chinese population (No: LZUKQ-2019-042). Finally, retrieving this number of cases isn’t doubtful especially in large population community as Chinese population.

The clinical application of these findings suggests that if SOS is fusing, the individual would have the maximum amount of mandibular growth; in total mandibular length, mandibular body length, mandibular width, mandibular height, and mandibular ramal height. So, these findings may be useful in the three-dimensional growth prediction of the mandible during treatment planning for orthodontic, dentofacial orthopedic, or orthognathic surgery for both sexes.

Regarding the clinical implications and questionability of using the SOS fusion stages as a mandibular skeletal indicator; the study was designed to compare different categories of ages based on the expected mandibular growth changes during these different ages and because age is a weak determinant, a more standardized and well-established method was selected to answer this question (SOS) so that the clinician can decide whether to proceed with the growth modification mechanics. The use of SOS is considered as a reliable tool and correlated with other established methods; Hand-wrist maturation and CVM indices [10, 18, 22, 23, 25, 36, 45–49]. Finally, we didn’t absolutely judge the exact final mandibular size or the absolute residual growth which was difficult compared to the limited range of stages [22, 24, 36].

In this study, it is worthy to mention that regarding the new information added to the orthodontic literature, we gave a view about three-dimensional mandibular growth during SOS fusion stages in both sexes, assessing the reliability of the SOS method as a skeletal indicator of 3D mandibular growth, and 3D mandibular growth spurt based on SOS fusion stages, which aren’t available in the published in the literature. This may have important for the orthodontist in diagnosis or considered as a base for further research in the future.

The limitation of this study starts with its nature as a cross-sectional study; there is no doubt that the longitudinal studies of mandibular growth and development provide a more thorough understanding. However, the challenges of acquiring high sample numbers for a longitudinal study, the related increase in the number of radiographic exposures, and the ethical considerations are likely to rule out this approach and use the cross-sectional direction. So, the cross-sectional design was selected based on similar published study about maxillary and mandibular growth [28]. Also, this study might be considered as primary reporting in this field, and we hope there more detailed studies in future of a longitudinal design. Another limitation is that the ethnic group is limited to the Chinese population, making it less practical for other ethnicities. The sample had no skeletal classes or facial pattern specifications, which might affect the current findings. Detailed description of the SOS method can only be done with CBCT imaging, and it is not very obvious in plain x- ray.

## Conclusions

The SOS maturation stages suggested to be a valid and reliable craniofacial skeletal indicator as evaluated and compared with three-dimensional cephalometric measurements of the mandibular in both sexes.

There was no sexual dimorphism regarding SOS maturation stages for total mandibular length and mandibular body width, while had sexual dimorphism for mandibular height, inter-gonial, and inter-condylar width.

The growth increments between SOS stages 2 and 3 were higher than those between stages 1 and 2 and staged 3 and 4 in most of the three-dimensional cephalometric dimensions of the mandible in both sexes.

The growth curves showed high active growth of the mandible as the SOS was still fusing (especially stages 2 and 3) than those of the fused (stage 4). Moreover, growth acceleration occurred earlier in females than males regarding chronological age but not for SOS maturation stages.

## Supplementary Information

**Additional file 1: Table S1.** Definitions of the three-dimensional skeletal landmarks of the mandible; **Table S2.** Definitions of the three-dimensional craniofacial reference planes; **Table S3.** Inter and intra-observer SOS staging reliability; and **Table S4.** Reliability analysis of three-dimensional mandibular measurements.

**Additional file 2**: The raw data of 3D mandibular measurements across SOS fusion stages.

## Data Availability

All data and materials are available in the Orthodontics Department of stomatology school, Lanzhou University, China. Please get in touch with the corresponding author for any requests.

## Abbreviations

SOS: Spheno-occipital synchondrosis
DICOM: Digital Imaging and Communications in Medicine
CBCT: Cone beam computed tomography
CT: Computed tomography
3D: Three dimensional
ICC: Intra-class correlation coefficient

## References

[1] Ford EHR (1958). Growth of the human cranial base. Am J Orthod.

[2] Afrand M, Oh H, Flores-Mir C, Lagravere-Vich MO: Growth changes in the anterior and middle cranial bases assessed with cone-beam computed tomography in adolescents. *Am J Orthod Dentofacial Orthop* 2017, 151(2):342–50. 10.1016/j.ajodo.2016.02.032 28153164

[3] Goldstein JA, Paliga JT, Wink JD, Bartlett SP, Nah H-D, Taylor JA (2014). Earlier evidence of spheno-occipital synchondrosis fusion correlates with severity of midface hypoplasia in patients with syndromic craniosynostosis. Plast Reconstr Surg.

[4] Adem C, Lafitte F, Jarquin S, Guillem P, Chiras J (1999). The persistence of a spheno-occipital synchondrosis in an adult. J Radiol.

[5] Lewis AB, Roche AF (1974). Cranial base elongation in boys during pubescence. Angle Orthod.

[6] Krieg WL (1987). Early facial growth accelerations: a longitudinal study. Angle Orthod.

[7] Faltin KJ, Faltin RM, Baccetti T, Franchi L, Ghiozzi B, McNamara JA (2003). Long-term effectiveness and treatment timing for Bionator therapy. Angle Orthod.

[8] Baccetti T, Franchi L, Toth LR, McNamara JA (2000). Treatment timing for Twin-block therapy. Am J Orthod Dentofac Orthop.

[9] Farman AG, Scarfe WC (2009). The basics of maxillofacial cone beam computed tomography. Semin Orthod.

[10] Fernandez-Perez MJ, Alarcon JA, McNamara JA, Velasco-Torres M, Benavides E, Galindo-Moreno P, Catena A (2016). Spheno-occipital synchondrosis fusion correlates with cervical vertebrae maturation. PLoS ONE.

[11] Ferrillo M, Curci C, Roccuzzo A, Migliario M, Invernizzi M, de Sire A (2021). Reliability of cervical vertebral maturation compared to hand-wrist for skeletal maturation assessment in growing subjects: A systematic review. J Back Musculoskelet Rehabil.

[12] Gabriel DB, Southard KA, Qian F, Marshall SD, Franciscus RG, Southard TE. Cervical vertebrae maturation method: poor reproducibility. Am J Orthod Dentofacial Orthop. 2009;136(4):478 e471–477; discussion 478–480. 10.1016/j.ajodo.2007.08.028 19815136

[13] Chen F, Terada K, Hanada K (2004). A new method of predicting mandibular length increment on the basis of cervical vertebrae. Angle Orthod.

[14] Nestman TS, Marshall SD, Qian F, Holton N, Franciscus RG, Southard TE (2011). Cervical vertebrae maturation method morphologic criteria: poor reproducibility. Am J Orthod Dentofacial Orthop.

[15] Gray S, Bennani H, Kieser JA, Farella M (2016). Morphometric analysis of cervical vertebrae in relation to mandibular growth. Am J Orthod Dentofacial Orthop.

[16] Engel TP, Renkema AM, Katsaros C, Pazera P, Pandis N, Fudalej PS (2016). The cervical vertebrae maturation (CVM) method cannot predict craniofacial growth in girls with Class II malocclusion. Eur J Orthod.

[17] Grave KC, Brown T (1976). Skeletal ossification and the adolescent growth spurt. Am J Orthod.

[18] Baccetti T, Franchi L, McNamara JA (2005). The cervical vertebral maturation (CVM) method for the assessment of optimal treatment timing in dentofacial orthopedics. Semin Orthod.

[19] Simpson SW, Kunos CA (1998). A radiographic study of the development of the human mandibular dentition. J Hum Evol.

[20] Mahdian A, Kavousinejad S, Dashti M, Behnaz M. A systematic review of methods to determine skeletal maturation based on cervical vertebrae. J Regen Reconstruct Restor (Triple R), 3(1).

[21] Honda K, Bjørnland T (2006). Image-guided puncture technique for the superior temporomandibular joint space: value of cone beam computed tomography (CBCT). Oral Surg Oral Med Oral Pathol Oral Radiol Endod.

[22] Jabour AS. Assessment of spheno-occipital synchondrosis fusion timing and an evaluation of its relationship with skeletal maturity, dental maturity and mandibular growth. Diss. US.: Case Western Reserve University; 2017.

[23] Dillon ME. Comparison of spheno-occipital synchondrosis closure, cervical vertebrae maturation and hand-wrist maturation as skeletal maturation indicators. M.S. Ann Arbor, US: University of Minnesota; 2018.

[24] Alhazmi A, Vargas E, Palomo JM, Hans M, Latimer B, Simpson S (2017). Timing and rate of spheno-occipital synchondrosis closure and its relationship to puberty. PLoS ONE.

[25] Demirturk Kocasarac H, Altan AB, Yerlikaya C, Sinanoglu A, Noujeim M (2017). Correlation between spheno-occipital synchondrosis, dental age, chronological age and cervical vertebrae maturation in Turkish population: is there a link?. Acta Odontol Scand.

[26] Al-Gumaei WS, Al-Attab R, Alhammadi MS, Al-Rokhami RK, Almashraqi AA, Zhenlin G, Abdulghani EA, Zheng Y (2022). Evaluation of spheno-occipital synchondrosis fusion in Chinese population using CBCT: a cross-sectional study. JCDP.

[27] Fortanely BE (2020). The influence of the spheno occipital synchondrosis fusion on craniofacial form.

[28] Manabe A, Ishida T, Kanda E, Ono T (2022). Evaluation of maxillary and mandibular growth patterns with cephalometric analysis based on cervical vertebral maturation: a Japanese cross-sectional study. PLoS ONE.

[29] Franklin D, Flavel A (2014). Brief communication: timing of spheno-occipital closure in modern Western Australians. Am J Phys Anthropol.

[30] Hisham S, Flavel A, Abdullah N, Noor MHM, Franklin D (2018). Quantification of spheno-occipital synchondrosis fusion in a contemporary Malaysian population. Forensic Sci Int.

[31] Sinanoglu A, Kocasarac HD, Noujeim M (2016). Age estimation by an analysis of spheno-occipital synchondrosis using cone-beam computed tomography. Leg Med.

[32] Vale F, Francisco I, Lucas A, Roseiro A, Caramelo F, Sobral A. Timing of spheno-occipital synchondrosis ossification in children and adolescents with cleft lip and palate: a retrospective case-control study. Int J Environ Res Public Health. 2020;17(23). 10.3390/ijerph17238889 PMC7731241 33260492

[33] Alhammadi MS, Al-mashraqi AA, Alnami RH, Ashqar NM, Alamir OH, Halboub E, Reda R, Testarelli L, Patil S (2021). Accuracy and reproducibility of facial measurements of digital photographs and wrapped cone beam computed tomography (CBCT) photographs. Diagnostics.

[34] Lottering N, MacGregor DM, Alston CL, Gregory LS (2015). Ontogeny of the spheno-occipital synchondrosis in a modern Queensland, Australian population using computed tomography. Am J Phys Anthropol.

[35] Shirley NR, Jantz RL (2011). Spheno-occipital synchondrosis fusion in modern Americans. J Forensic Sci.

[36] Alhazmi A, Aldossary M, Palomo JM, Hans M, Latimer B, Simpson S (2021). Correlation of spheno-occipital synchondrosis fusion stages with a hand-wrist skeletal maturity index: a cone beam computed tomography study. Angle Orthod.

[37] Bassed RB, Briggs C, Drummer OH (2010). Analysis of time of closure of the spheno-occipital synchondrosis using computed tomography. Forensic Sci Int.

[38] Baker MJ (2019). Analysis of spheno-occipital synchondrosis (SOS) fusion in a contemporary southern Nevada subadult Hispanic population using archival cone-beam computerized tomography (CBCT) images.

[39] Ok G, Sen Yilmaz B, Aksoy DO, Kucukkeles N (2021). Maturity evaluation of orthodontically important anatomic structures with computed tomography. Eur J Orthod.

[40] Okamoto K, Ito J, Tokiguchi S, Furusawa T (1996). High-resolution CT findings in the development of the sphenooccipital synchondrosis. AJNR Am J Neuroradio.

[41] Golchini E, Rasoolijazi H, Momeni F, Shafaat P, Ahadi R, Jafarabadi MA, Rahimian S (2020). Investigation of the relationship between mandibular morphology and upper airway dimensions. J Craniofac Surg.

[42] Wang RH, Ho C-T, Lin H-H, Lo L-J. Three-dimensional cephalometry for orthognathic planning: Normative data and analyses. J Formos Med Assoc. 2020;119(1, Part 2):191–203. 10.1016/j.jfma.2019.04.001 31003919

[43] Park J, Baumrind S, Curry S, Carlson SK, Boyd RL, Oh H (2019). Reliability of 3D dental and skeletal landmarks on CBCT images. Angle Orthod.

[44] Sato K, Mito T, Mitani H (2001). An accurate method of predicting mandibular growth potential based on bone maturity. Am J Orthod Dentofacial Orthop.

[45] Uysal T, Ramoglu SI, Basciftci FA, Sari Z (2006). Chronologic age and skeletal maturation of the cervical vertebrae and hand-wrist: is there a relationship?. Am J Orthod Dentofacial Orthop.

[46] Lai EH, Liu JP, Chang JZ, Tsai SJ, Yao CC, Chen MH, Chen YJ, Lin CP (2008). Radiographic assessment of skeletal maturation stages for orthodontic patients: hand-wrist bones or cervical vertebrae?. J Formos Med Assoc.

[47] Hassel B, Farman AG (1995). Skeletal maturation evaluation using cervical vertebrae. Am J Orthod Dentofacial Orthop.

[48] O'Reilly MT, Yanniello GJ (1988). Mandibular growth changes and maturation of cervical vertebrae: a longitudinal cephalometric study. Angle Orthod.

[49] Alhazmi A, Aldossary M, Palomo JM, Hans M, Latimer B, Simpson S (2021). Correlation of spheno-occipital synchondrosis fusion stages with a hand-wrist skeletal maturity index: a cone beam computed tomography study. Angle Orthod.

[50] Powell TV, Brodie AG (1963). Closure of the spheno-occipital synchondrosis. Anat Rec.

[51] Singh GD, McNamara JA, Lozanoff S (1997). Morphometry of the cranial base in subjects with Class III malocclusion. J Dent Res.

[52] Enlow D. Facial growth and development in the rhesus monkey. In: *Handbook of Facial Gowth.* 3rd edn. Philadelphia: WB Saunders Company; 1990: 444–54.

[53] Graf CC, Dritsas K, Ghamri M, Gkantidis N. Reliability of cephalometric superimposition for the assessment of craniofacial changes: a systematic review. Eur J Orthod 2022:cjab082. 10.1093/ejo/cjab082 35175333

[54] Law CJ, Baliga VB, Tinker MT, Mehta RS (2017). Asynchrony in craniomandibular development and growth in Enhydra lutris nereis (Carnivora: Mustelidae): are southern sea otters born to bite?. Biol J Linn Soc.

[55] Dibbets JM, Trotman CA, McNamara JA, van der Weele LT, Janosky JE (1997). Multiple linear regression as an analytical tool in cephalometric studies. Br J Orthod.

