# Supplementary material for: Comparison of spheno-occipital synchondrosis maturation stages with three-dimensional assessment of mandibular growth
Source: BMC Oral Health. 2022 Dec 30;22:654. doi: 10.1186/s12903-022-02692-3 (PMC9801555; doi:10.1186/s12903-022-02692-3)
Supplement: Supplementary file 1 — Additional file 1: Table S1. Definitions of the three-dimensional skeletal landmarks of the mandible; Table S2. Definitions of the three-dimensional craniofacial reference planes; Table S3. Inter and intra-observer SOS staging reliability; and Table S4. Reliability analysis of three-dimensional mandibular measurements. [file 12903_2022_2692_MOESM1_ESM.docx]

**Supplementary table 1.** Definitions of the three-dimensional skeletal landmarks of the mandible.

| **Landmark** | **Definition** |
| --- | --- |
| **Orientation landmarks:** | |
| Basion (Ba) | The most anterior and inferior point of the foramen magnum. |
| Nasion (N) | The midpoint of the frontonasal sutures. |
| Porion (Po) | The most superior point of the external auditory meatus. |
| Orbitale (Or) | The most inferior point of infra-orbital rim |
| **Mandibular landmarks:** | |
| Gnathion (Gn) | The most anterior and inferior point on the contour of the mandibular symphysis. |
| Menton (Me) | The most inferior midpoint of the chin on the outline of the mandibular symphysis. |
| Gonion (Go-mid) | The midpoint of the right and left condylion point: is dropping perpendicular from the intersection point of the tangent lines to the posterior margin of the mandibular vertical ramus and inferior margin of the mandibular body or horizontal ramus. |
| Condylion (Co-mid) | The midpoint of the right and left condylion point: is the most postero-superior point of each mandibular condyle in the sagittal plane. |
| Antegonion (Ag) | The deepest point of the antegonial depression. |

**Supplementary table 2.** Definitions of the three-dimensional craniofacial reference planes.

| Plane | Definition |
| --- | --- |
| Frankfurt horizontal plane (FHP) | The horizontal plane (X) was passing through the right and left porions and the right orbitale. |
| Mid-sagittal plane (MSP) | The mid-sagittal plane (Y) was passing through nasion and basion and perpendicular to the FH plane. |
| Coronal plane (CP) | The coronal plane (Z) was passing through sella turcica and perpendicular to the midsagittal plane and FH plane. |
| Mandibular plane (MP) | The mandibular plane is deﬁned by a plane that passes the menton and midpoint of Gonion (Go mid – Me) landmarks |

**Supplementary table 3.** Inter and intra-observer SOS staging reliability.

|  | Intra-observers | | Interobservers |
| --- | --- | --- | --- |
|  | W.A. | R.A. | Both |
| Number of cases | 100 | 100 | 500 |
| Weighted Kappa (k) | 0.922 | .922 | 0.900 |
| Significance | P < 0.001 | P < 0.001 | P < 0.001 |

**Supplementary table 4.** Reliability analysis of three-dimensional mandibular measurements.

| **Measurements** | **Inter-observer reliability** | | | | **Intra-observer reliability** | | | |
| --- | --- | --- | --- | --- | --- | --- | --- | --- |
|  | **ICC** | **TEM** | **RTEM** | **R** | **ICC** | **TEM** | **RTEM** | **R** |
| Co mid –Gn (mm) | 0.9988 | 0.3559 | 0.1525 | 0.9945 | 0.9997 | 0.1997 | 0.0855 | 0.9982 |
| Go mid-Me (mm) | 0.9924 | 0.7058 | 0.4946 | 0.9666 | 0.9962 | 0.4961 | 0.3476 | 0.9830 |
| Co mid -Go mid (mm) | 0.9939 | 0.5703 | 0.4854 | 0.9731 | 0.9997 | 0.1403 | 0.1195 | 0.9983 |
| Ag-Ag (mm) | 0.9860 | 0.9309 | 0.5090 | 0.9327 | 0.9996 | 0.1546 | 0.0844 | 0.9982 |
| Go-Go (mm) | 0.9969 | 0.5549 | 0.2744 | 0.9843 | 0.9978 | 0.4416 | 0.2186 | 0.9899 |
| Co-Co (mm) | 0.9959 | 0.6585 | 0.3001 | 0.9816 | 0.9987 | 0.3567 | 0.1626 | 0.9944 |
| (Ag / FHP) ° | 0.9972 | 0.3524 | 0.5618 | 0.9853 | 0.9986 | 0.2403 | 0.3816 | 0.9931 |
| (Go mid-Me /FHP.) ° | 0.9976 | 0.5045 | 0.7991 | 0.9883 | 0.9991 | 0.3171 | 0.5018 | 0.9954 |
| (Ag-Ag / FHP) ° | 0.9806 | 0.1722 | 8.6992 | 0.9152 | 0.9894 | 0.1243 | 6.3273 | 0.9527 |

Note: TEM and rTEM indicate an absolute and relative technical error of measurement. ICC indicates the interclass correlation. All R* values were higher than the 0.95 percent indicated cut-off.

**Supplementary tables legends:**

**Supplementary table 1.** Definitions of the three-dimensional skeletal landmarks of the mandible.

**Supplementary table 2.** Definitions of the three-dimensional craniofacial reference planes.

**Supplementary table 3.** Inter and intra-observer SOS staging reliability.

**Supplementary table 4.** Reliability analysis of three-dimensional mandibular measurements.
